# Comparison between D‐dimer levels in diabetic and non‐diabetic positive COVID‐19 adult patients: A hospital‐based study

**DOI:** 10.1002/edm2.349

**Published:** 2022-05-25

**Authors:** Ibrahim Hashim Ibrahim Elbashir, Hala Kamal Ali Mohamed, Mohammed Elmujtba Adam Essa, Ahmed Seri

**Affiliations:** ^1^ Department of Clinical Immunology Sudan Medical Specialization Board (SMSB) Khartoum Sudan; ^2^ Departments of Microbiology, Faculty of Medicine African International University (AIU) Khartoum Sudan; ^3^ Faculty of Medicine Sudan University of Science and Technology Khartoum Sudan; ^4^ Faculty of Medicine Alfashir University Alfashir Sudan; ^5^ Departments of Clinical Medicine Medical and Cancer Research Institute Nyala Sudan; ^6^ Department of Clinical Immunology Royal Care International Hospital Khartoum Sudan

**Keywords:** COVID‐19, D‐dimer, diabetes, ICU, mortality

## Abstract

**Background:**

Diabetes is one of the most common associated comorbidity with severe acute respiratory syndrome coronavirus (SARS‐CoV‐2) pneumonia patients.

Coagulation disorders with D‐dimer levels are increased in both diseases. This study aimed to compare the levels of D‐dimer in DM and non‐DM patients with coronavirus disease (COVID‐19) and correlate it with the disease severity.

**Methods:**

This is a cross‐sectional hospital‐based study. It was conducted at royal care hospital, isolation COVID‐19 Centre in 2021. The study included 130 patients with COVID‐19 who fulfilled the inclusion criteria. Data were collected through a structured datasheet. The disease was diagnosed by a nasal swab polymerase chain reaction (PCR) Participants were divided into diabetics and non‐diabetics depending on the history from the datasheet. The data were analysed with Statistical Package of Social Science (SPSS) version 23.

**Results:**

The study showed that 73.1% of the participants were males and 26.9% were females. The most frequent age group was >65 years. The percentages of diabetics and non‐diabetics, among the study participants, were found to be 41.5% and 58.5%, respectively. Moreover, 52.3% were admitted to the intensive care unit (ICU). This study revealed that D‐dimer was higher compared with diabetes mellitus. The diabetics were associated with higher levels of D‐dimer compared to non‐diabetics. Regarding the correlation between the level of D‐dimer and severity of COVID‐19, it was found that there is a significant association, as the ICU patients were associated with higher levels of D‐dimer in comparison with non‐ICU patients.

**Conclusion:**

This study concluded that there is a significant association between the high D‐dimer level and severity of COVID‐19 among diabetic patients.

## Graphical abstract

Shows the age distributions between the patients.

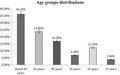

## 1 INTRODUCTION

COVID‐19 is caused by SARS‐CoV‐2.^1^, ^2^ The spread and severity of SARS‐CoV‐2 infection among individuals can be linked to health status and exposure. Several reasons for this have been highlighted globally and may be responsible for the extent and severity of COVID‐19 during the last year.^3^ It is well recognized that the extent and severity of the infection are related to diabetic status. Some of the potential mechanisms explaining this situation are altered immune response, cytokine storm and glucose metabolism.^4^

Diabetes patients have a greater risk of getting various infections. So far, reports concluded that DM has been one of the high‐risk comorbidities in COVID‐19 patients, which may be due to other risk factors such as hypertension, age and obesity, associated with diabetes. It may also be because there are intrinsic factors in DM patients that make them more vulnerable to the cytokine storm. In addition, as a result, this condition may consequence from an imbalance between fibrinolysis and coagulation factors, which may be responsible for thrombotic events, leading to death.^5^, ^6^, ^7^, ^8^ D‐dimer, a fibrin degradation product, is widely used as a biomarker of thromboembolism for critical cases and as a prognostic marker. Thus, as a biomarker to predict the significance and the risk of the disease, D‐dimer has been considered because COVID‐19 is a pro‐coagulant state.^9^, ^10^ Many laboratory markers can predict the severity of COVID‐19; those are mainly associated with poor outcomes including LDH, CRP, high D‐dimer and high‐sensitivity cardiac troponin I.^11^, ^12^

Since DM was established as a predictor of the seriousness of COVID‐19, it remains to be seen why D‐dimer values vary between those with and without diabetes until they can determine that this was one of the factors leading to extreme COVID‐19 disease, which increased the vulnerability to thromboembolic events. Therefore, this study aims to compare the levels of D‐dimer in diabetic and non‐diabetic patients with COVID‐19, and a link between D‐dimer and mortality disease among the patients.

### 1.1 Problem statement

COVID‐19 can lead to devastating morbidity and mortality; people with diabetes have a higher risk of getting various infections. So far, studies have shown that diabetes has been one of the serious comorbidities in patients with COVID‐19; this may be due to other risk factors, such as age, hypertension and obesity, associated with diabetes. D‐dimer is commonly elevated in patients with COVID‐19. D‐dimer levels correlate with disease severity and are a reliable prognostic marker for in‐hospital mortality in patients admitted for COVID‐19.

### 1.2 Justification

D‐dimer assessment has reliable parameters for evaluating the prognosis in COVID‐19 patients associated with diabetes at the time of diagnosis, which may help us at an early stage to predict the severity of the disease and differentiate high‐risk and low‐risk patients, thus might help in prioritizing hospital admission and saving medical resources and will help us to make an early intervention, and this may lead to slow the progression of the diseases and making optimal treatment decisions.

## 2 METHODOLOGY

### 2.1 Study design

A cross‐sectional hospital‐based study.

### 2.2 Study area

This study was conducted in isolation at COVID‐19 centre, royal care hospital, Khartoum, Sudan.

### 2.3 Study population

All patients with COVID‐19 infection were diagnosed by PCR, and admitted to the hospital from May 2021 to July 2021.

### 2.4 Sample Size

On hundred thirty participants were included in the study. This number comprised the total number of patients who met inclusion criteria during the study period.

### 2.5 Data collection tools

The data were collected by cheek list, using electronic medical records. The data included age, gender, medical history, comorbidity with diabetics, ward and ICU admission. D‐dimer was the only laboratory finding obtained from hospital records.

### 2.6 Data analysis

Data were analysed by using the statistical package of social sciences (SPSS) version 23 and tested by using the chi‐square test. Yielded results were considered statistically significant at a *p* value <.05. The severity of COVID‐19 was categorized as per (WHO care bundle)(39): non‐critical (non‐ICU admission)/ critical (ICU admission) entities.

## 3 RESULTS

**FIGURE 1:**
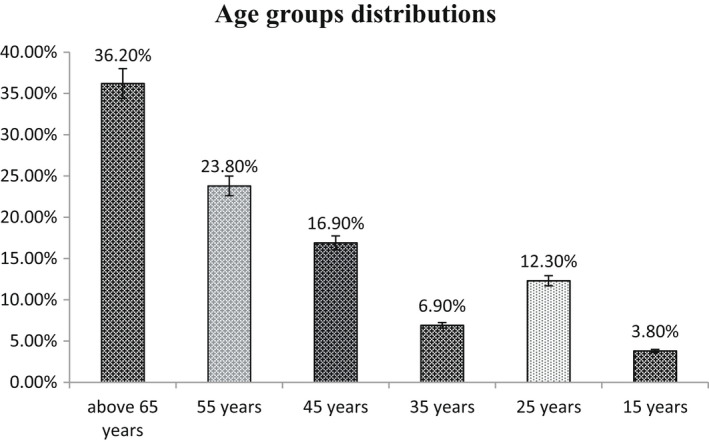
Age distributions between the patients

The age group above 65 years is the most frequent group with 36.2% of all the patients, followed by the age group of 55, 45, 25, 35 and 15 years, respectively, as follows: 23.8%, 16.9%, 12.3%, 6.9% and 3.8% (Figure 1). Regarding the gender of the patients, males were in the majority 73.1% (*n* = 95), as compared to the females 26.9% (*n* = 35). The percentages of diabetics and non‐diabetics, among the study participants, were found to be 41.5% and 58.5%, respectively. Among all the patients with COVID‐19, 51.5% were Admitted‐ICU and 48.5 were Not‐Admitted‐ICU. Most of the patients have high D‐dimer 67.7%, while the remaining 32.2% have normal D‐dimer.

The association between D‐dimer level and comorbidity with diabetes mellitus is found to be significantly correlated, as the diabetics were associated with higher levels of D‐dimer compared with non‐diabetics, as shown in (Table 1.). Regarding the correlation between the level of D‐dimer and severity of COVID‐19, it was found that there is a significant association, as the ICU patients were associated with higher levels of D‐dimer in comparison with non‐ICU patients, as shown in (Table 2). In the correlation between the presence of diabetes as comorbidity and the severity of COVID‐19, it was found that the ICU patients were associated with the diabetic group in comparison with non‐ICU patients. As shown in (Table 3).

**TABLE 1:** Relationship between the D‐dimer level and comorbidity with diabetes mellitus

	Normal level of D‐dimer	High level of D‐dimer	*p* value*
	*N*	*N*	
Diabetics	12	42	.033
Non‐diabetics	30	46	

^*^Statistically significant when *p* value <.05.

**TABLE 2:** Relationship between the D‐dimer level and severity of COVID‐19

	Normal level D‐dimer	High level of D‐dimer	*p* value*
	*N*	*N*	
ICU	10	58	.00001
Non‐ICU	32	30	

*Statistically significant when *p* value <.05.

**TABLE 3:** Relationship between the presence of Diabetes as comorbidity and severity of COVID‐19

	Non‐diabetics	Diabetics	*p* value*
	*N*	*N*	
ICU	32	36	.024
Non‐ICU	44	18	

^*^Statistically significant when *p* value <.05.

## 4 DISCUSSION

Since the start of the COVID pandemic in 2019, DM considered one of the major comorbidities encountered in severe forms of COVID‐19. Many reports showed that the prevalence of DM was nearly 10%, and his incidence in severe cases was about twofold that of non‐severe patients.^13^ D‐dimer is a marker of fibrin and fibrinolysis turnover, with unique molecular properties to work as a biological marker for haematological abnormalities.^14^, ^15^ Hyperglycaemia in diabetes is thought to cause dysfunction of the immune response, in many ways such as altering macrophages functions and deregulating neutrophil synthesis, which may lead to failure to control the spread of invading pathogens in diabetic subjects. Therefore, diabetic subjects are known to be more susceptible to infections.^16^ In our study, we discovered that 77.7% of all the studied patients' diabetic patients have a high level of D‐dimer and 67% of all the patients, this may suggest that they were more probable than non‐diabetic patients have high risk of coagulopathy. This can explain that hyperglycaemia can cause a pro‐thrombotic status,^17^ according to two distinct pathways: the non‐glycation and the oxidative stress, which improve thrombin production during acute hyperglycaemia. Also, enzymatic reduces the function of heparin co‐factor II and antithrombin III.^18^ Thus, persistent hyperglycaemia can lead to endothelial dysfunction and inflammation, contributing to thrombus formation. In conclusion, hyperglycaemia may contribute to the formation of thrombi due to an imbalance between anticoagulation, pro coagulation and fibrinolysis.^17^, ^19^

In the association between the D‐dimer level and the severity of COVID‐19, it was found that the higher D‐dimer level was associated with higher disease severity (i.e. ICU patients); this was approximate to what had been concluded by Mishra et al.^6^ According to the Boli et al. report, the prevalence of diabetes in intensive care units was twice as high in COVID‐19 patients.^20^ In this study, we found that 52.3% of all the patients were hospitalized in ICU admission; among those patients, 53% are diabetic patients. In addition, another study conducted in Wuhan found that 22% of patients with DM had an increased risk of admission to ICU, being on mechanical ventilation and death, where only non‐diabetic patients reported 6% of the dietetic risks and complications with COVID‐19.^21^ Overall, high D‐dimer and DM are indicators of a poor prognosis. Still, the combination of the two will be worsening and aggravates the prognosis with progression to high‐risk complications and death, which requires proper therapeutic treatment and strict supervision. In addition, compared with non‐diabetic patients, it is found that some inflammation‐related biomarkers are elevated in diabetic patients, this can also be attributed to diabetic patients who already have baseline low‐grade chronic inflammation, and their innate and adaptive immune systems are deregulated, making them more vulnerable to the cytokine storm, responsible for the rapid deterioration and poor prognosis of COVID‐19.

Regarding the sex of the patients group, males represent 73.1% of all the study patients, while the remaining 26.9% were females. Other reports showed a similar results.^22^ The higher incidence of male patients compared with females can be related to many factors including the biological differences in the immune systems of both sex in general, females are more resistant to infections than males, and this is maybe mediated by factors including sex hormones and high expression of coronavirus receptors (ACE 2) in males, although the lifestyle, such as higher levels of smoking and drinking among men as compared to women, can play role in their immune system.^23^ Concerning the age distribution, it was found that a parallel correlation between age and COVID‐19 affection as more than a third of the patients was above 65 years, and the same was observed in other studies where elderly people have a higher risk to be affected by COVID‐19.^13^

In conclusion, DM is the commonest comorbidity in COVID‐19 patients, and D‐dimer level and the inflammatory assessment have reliable parameters for assessing and evaluating the prognosis of the COVID‐19 patients as well as an accurate and practical coagulation parameter for predicting mortality. In our study, we found a significant correlation between D‐dimer with diabetes patients and the severity of COVID‐19. Male gender and old age were both linked to a high incidence of the disease.

## CONFLICT OF INTEREST

All authors declare that they have no conflict of interest and no funds have been received.

## ETHICAL APPROVAL

Ethics approval for this study was obtained from the Sudan Medical Specialization Board.

## AUTHOR CONTRIBUTIONS

Data collection, statistical analysis, manuscript writing by IHIE, MEAE, ASI. Supervision, critical revision and final editing by HKAM.

## CONSENT FOR PUBLICATION

Obtained.

## Data Availability

All the data used in the study are available from the first and corresponding author on reasonable request
